# Analysis and Evaluation of the Flagellin Activity of *Bacillus amyloliquefaciens* Ba168 Antimicrobial Proteins against *Penicillium expansum*

**DOI:** 10.3390/molecules27134259

**Published:** 2022-07-01

**Authors:** Meihuan Lu, Yahan Chen, Lijun Li, Yinghui Ma, Zefang Tong, Dongsheng Guo, Pingping Sun, Derong An

**Affiliations:** 1 Microbiology Institute of Shaanxi, Xi’an 710043, China; lu_meihuan@sina.com (M.L.); lijun_lli@163.com (L.L.); yinghuima@163.com (Y.M.); wawghqb@163.com (Z.T.); 2 College of Plant Protection and Shaanxi Key Laboratory of Molecular Biology for Agriculture, Northwest A&F University, Yangling 712100, China; yhchen1018@nwafu.edu.cn (Y.C.); gds1995908@163.com (D.G.); 3 College of Plant Protection, Gansu Agricultural University, Lanzhou 730070, China; 4 College of Horticulture and Plant Protection, Inner Mongolia Agricultural University, Huhhot 010018, China

**Keywords:** *Bacillus amyloliquefaciens*, antimicrobial protein, flagellin, *Penicillium expansum*

## Abstract

Blue mold caused by *Penicillium expansum* is one of the most common apple diseases, and it is becoming a serious threat in apple production. The strain *Bacillus amyloliquefaciens* Ba168 showed high levels of antimicrobial activity in our previous study. To analyze the antimicrobial protein of Ba168, a high-resolution LC-MS/MS proteomic analysis was performed. A total of 1155 proteins were identified from 5233 unique peptides. A total of 16 potential antimicrobial-activity-related proteins were identified; 10 of these proteins have direct antimicrobial effects, while 6 of these proteins are associated with the formation of antimicrobial substances. Then, an antifungal protein of Ba168 was isolated and purified by the sequential chromatography of DEAE Bio-sep FF anion exchange and Sephadex G-75. The single protein, named BP8-2, showed antifungal activity towards *Penicillium expansum*. The peptide mass fingerprinting of the protein band of BP8-2 had a high similarity with the amino acid sequences of flagellin protein. The results showed that BP8-2 significantly inhibited the growth of *P. expansum* and slowed the spread of apple blue mold. The results indicated that flagellin is one of the important antimicrobial substances from Ba168.

## 1. Introduction

Apple blue mold, caused by *Penicillium expansum*, is one of the most severe diseases, accounting for approximately 20% to 25% of economic loss during the postharvest storage and transport of fruits and vegetables [1]. Although people widely use chemical fungicides as an effective management strategy [2], sustainable and environmentally friendly strategies are required due to increasing environmental pollution, pesticide residues, and pesticide resistance [3]. Compared with the chemical synthetic fungicides of postharvest diseases, natural antagonistic microorganisms for biological control have emerged as a promising alternative [4]. Strains belonging to bacteria have been used as effective biocontrol agents (BCAs) for controlling multiple plant diseases [5,6,7], and bacillus-based productions comprise almost half of the commercially available biocontrol products all over the world [8]. In spite of much interest in the biocontrol of postharvest diseases, the biocontrol research of apple blue mold and its mechanisms is in the preliminary stage [9].

*Bacillusamyloliquefaciens* is an environmentally friendly Gram-positive bacterium, exhibiting rapid propagation and stress resistance [7,10]. The mechanisms of bacteriostatic inhibition involved in *B. amyloliquefaciens* include the production of antagonistic substances, the induction of host resistance, and competition between nutrition and growth space. The antagonistic substances produced by *B. amyloliquefaciens*, such as antibiotics, bacteriocin, cell-wall-degrading enzymes, and other antimicrobial proteins, are important in the prevention and inhibition of pathogens [11,12]. Lipopeptides (LPs) and polyketides are well-known antibiotics produced by *Bacillus* for plant protection, including surfactin, fengycin, iturin, difficidin, bacilysin, macrolactin, bacillaene, bacillomycin D, and bacillibactin [13]. However, there are still many secondary metabolites without separation and identification. 

In our previous study, *Bacillus amyloliquefaciens* Ba168 was isolated from the Qingling Mountains of China; it has a significant control effect on a variety of plant diseases, including bacteria, fungi, and viruses [14,15]. *B. amyloliquefaciens* Ba168 has the characteristics of strong stress resistance, fast growth, and good stability, and it has been used in the control of tomato bacterial wilt, wheat yellow mosaic disease, wheat take-all, etc. However, the antibacterial substance and its antibacterial mechanism in the Ba168 were not studied. To rapidly characterize the unidentified active metabolites in fermentation broth, the proteins secreted by Ba168 in liquid medium were first identified using liquid chromatography-tandem mass spectrometry (LC-MS/MS) analysis and identification. The antifungal protein was purified by column chromatography and identified through LC–MS/MS analysis.

## 2. Results

### 2.1. Identification of Antimicrobial Proteins in Ba168

In order to establish the protein expression profile of Ba168, proteomic analyses of Ba168 were performed using high-resolution LC-MS/MS. A total of 1155 proteins were identified with >95% confidence from 5233 unique peptides. A total of 16 potential antimicrobial-activity-related proteins were identified based on previous reports. In total, 10 of the identified proteins exhibited direct antimicrobial activity—namely, oxalate decarboxylase, a chitin-binding protein, flagellin, carboxypeptidase, aminopeptidase, an antimicrobial peptide, serine protease, subtilisin, leucine dehydrogenase, and β-glucanase. The other six proteins were associated with the formation of antimicrobial substances—namely, surfactin synthase, polyketide synthase, bacillomycin D synthetase C, bacillomycin D synthetase A, bacilysin biosynthesis protein, and antilisterial bacteriocin subtilosin biosynthesis protein (Table 1). The peptide mass spectra of eight proteins obtained by LC-MS/MS are shown in additional materials. The unique peptide sequences identified were as follows in: surfactin synthetase A, QAGCSADELSK (+MSn 583.2609 *m*/*z*); aminopeptidase, SSLPHGVASGK (+MSn 347.189 *m*/*z*); carboxypeptidase, HGYLAGEDR (+MSn 509.2411 *m*/*z*); polyketide synthase, AATDDSGNELPR (+MSn 623.289 *m*/*z*); chitin-binding protein, IASANGGSGQIDFGLDK (+MSn 825.4102 *m*/*z*); oxalate decarboxylase, TIASALVVVPGAMR (+MSn 757.4224 *m*/*z*); antimicrobial peptide, LVQSPNGNFAASFVLDGTK (+MSn 983.0076 *m*/*z*); flagellin, NAQDGISLIQTSEGALNETHSLLQR (+MSn 1348.1851 *m*/*z*).

### 2.2. Purification of the Antifungal Protein in Ba168

**Figure 1:**
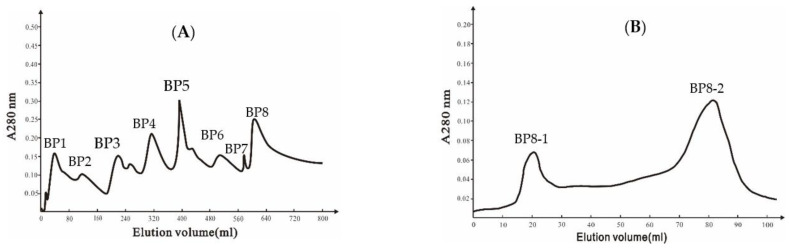
(**A**) Ion-exchange chromatography of DEAE Bio-sep FF resin of Ba168 proteins; (**B**) gel chromatography of SephadexG-75 column of active fraction (peak BP8 in Figure 1A).

A DEAE Bio-sep FF chromatography column was used to isolate extracellular proteins from strain Ba168, and eight peaks were collected (Figure 1A). The eighth peak (BP8) showed the highest inhibitory ability against *P. expansum*. The eighth peak was further separated by Sephadex G-75 chromatography column, and two peaks were collected (Figure 1B), and the second peak (BP8-2) showed inhibitory ability against *P. expansum*.

### 2.3. Inhibitory Effects of the Antifungal Protein BP8-2 on P. expansum In Vitro

**Figure 2:**
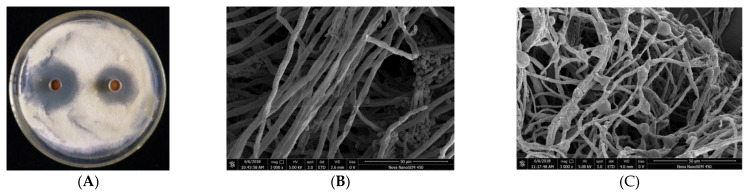
(**A**) Inhibition of *P. expansum* by protein BP8-2. (**B**) Untreated hyphae of *P. expansum* under SEM. (**C**) Treated hyphae of *P. expansum* under SEM.

The antifungal capacity of the protein BP8-2 was tested in vitro and in vivo, with apple blue mold (caused by *P. expansum*) as the test pathogen. The protein BP8-2 could significantly inhibit the growth of *P. expansum* in vitro (Figure 2A). As observed by scanning electron microscopy (SEM), the untreated hyphae of *P. expansum* were smooth and uniform, and distinct sporangia could be observed (Figure 2B), while *P. expansum* treated with protein BP8-2 showed enlarged hyphae, vesicle formation, distortion, or empty cells devoid of cytoplasm (Figure 2C), and no conidia or spores were found in the treated *P. expansum*. The MIC of the BP8-2 against *P. expansum* was 45 µg/mL. 

### 2.4. Control Effect of the Antifungal Protein BP8-2 on P. expansum In Vivo

**Figure 3:**
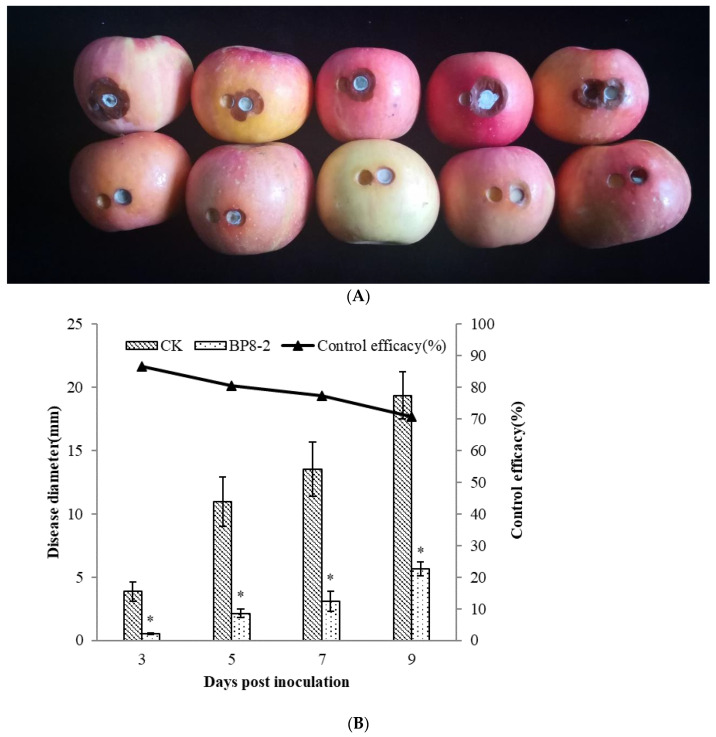
The control effect of protein BP8-2 on *P. expansum* in red Fuji apples (**A**) and its data analysis (**B**). Statistical analysis was performed using Dunnet’s comparative test. * *p* <0.05, compared to control.

In order to test the antifungal capacity of the protein BP8-2 in vivo, the control effect of protein BP8-2 against *P. expansum* in red Fuji apples was tested. Protein BP8-2 could significantly inhibit the spread of *P. expansum* on red Fuji apples (Figure 3A), and its control effect against *P. expansum* on red Fuji apples was 86.56%, 80.57%, 77.22%, and 70.85% within 3, 5, 7, and 9 days post inoculation, respectively (Figure 3B).

### 2.5. Identification of the Antifungal Protein BP8-2 and Amplification of Its Genomes

**Figure 4:**
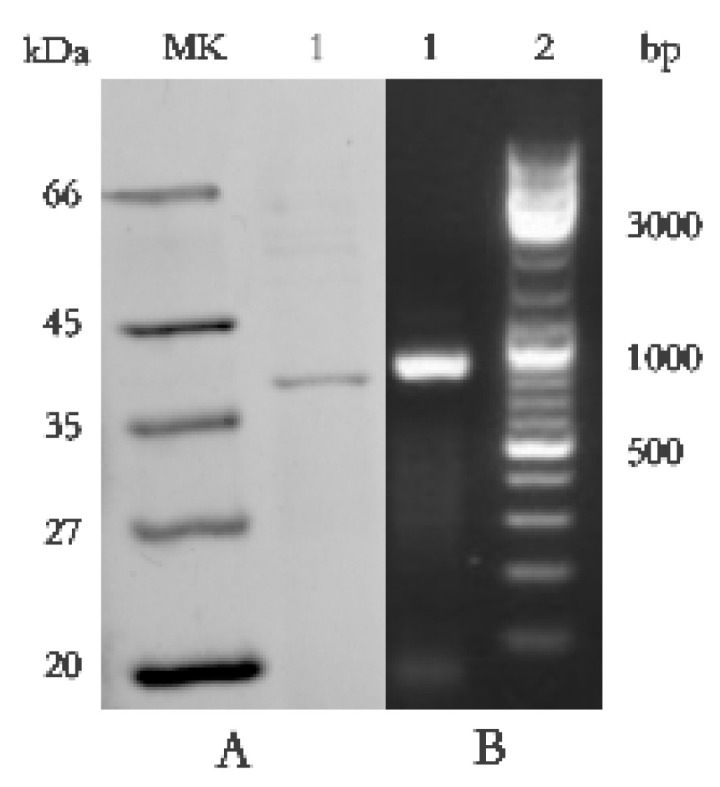
(**A**) Molecular weight and SDS-PAGE of the purified protein BP8-2. Lane MK, molecular mass stand; lane 1, purified protein BP8-2. (**B**) PCR amplification of hag gene corresponding to flagellin (line1).

**Figure 5:**
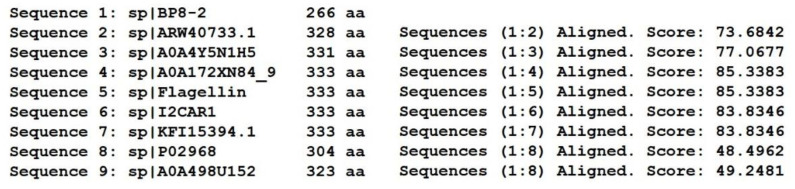

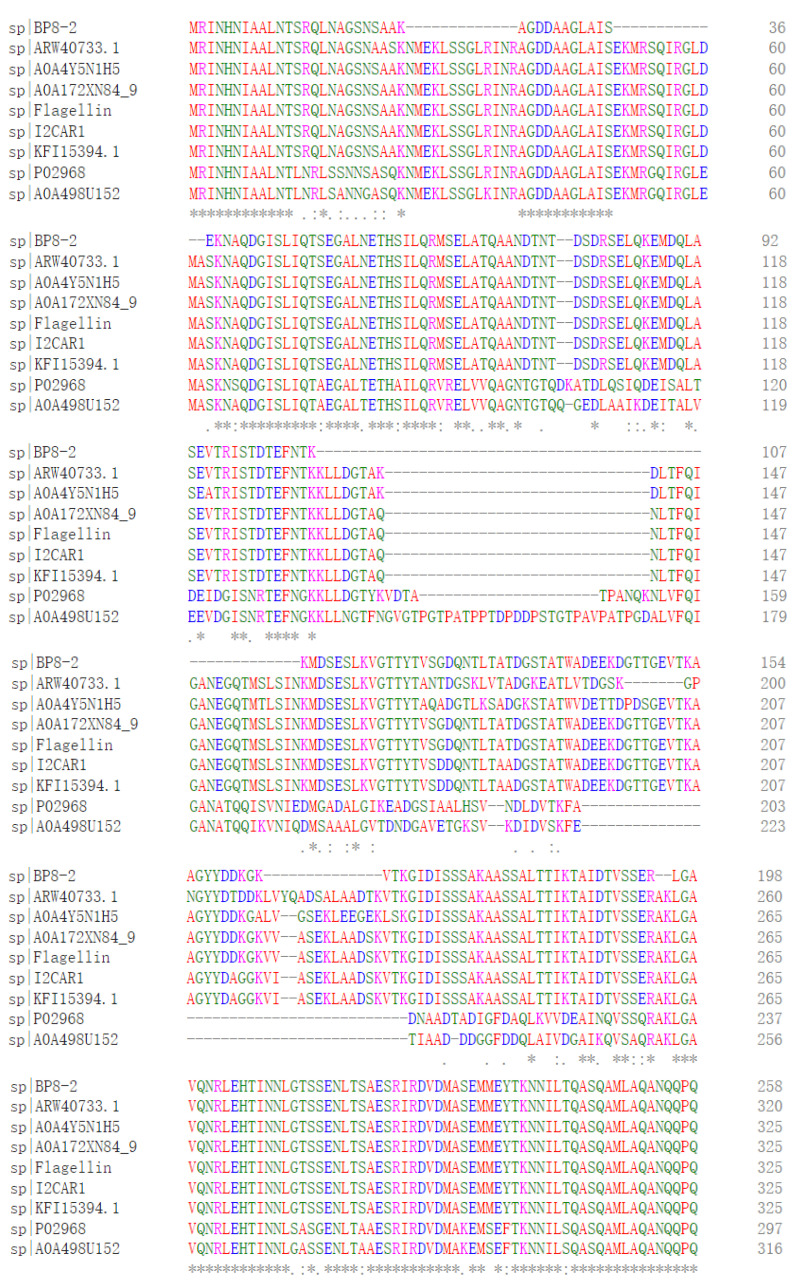

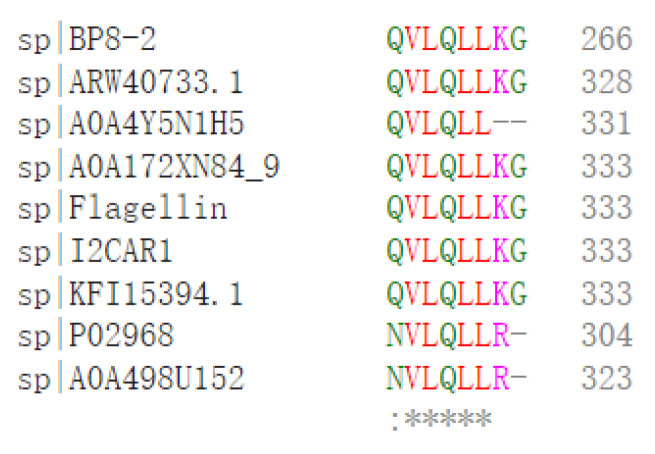
The protein BP8-2 peptide mapping finger sequences of Ba168 was compared with other flagellin proteins by the multiple sequence alignment analysis. Sequence 1~9: 1~BP8-2; 2~ARW40733.1 Flagellin (*B. amyloliquefaciens*); 3~A0A4Y5N1H5 Flagellin (*B. amyloliquefaciens*); 4~A0A172XN84_9 Flagellin (*B. velezensis*); 5~Flagellin (*B. amyloliquefaciens* Ba168); 6~I2CAR1 Flagellin (*B. amyloliquefaciens* Y2); 7~KFI15394.1 flagellin (*B. velezensis*); 8~P02968 Flagellin (*B. subtilis* strain 168); 9~A0A498U152 Flagellin (*B. safensis*). (* means the amino acid sequence is the same.)

SDS-PAGE detection indicated that BP8-2 was a single protein with a molecular weight of 35 kDa (Figure 4A). The band of BP8-2 protein was cut off, enzymolysized, and then analyzed by LC-MS/MS. Having compared it with the MASCOT database, the BP8-2 showed the highest matching rate to the flagellin protein of *Bacillus velezensis* (NCBI: A0A172XN84_9BACI, *p* < 0.05), with a molecular weight of 35 532 Da and containing 333 amino acid sequences. There were 28 peptide fragments that matched, including 266 amino acid residues, with a sequence coverage of 79.9%. The protein BP8-2 peptide mapping finger sequences of Ba168 was compared with other flagellin proteins by the multiple sequence alignment analysis in the ClustalW online program (Figure 5). The sequence of BP8-2 is the most similar to the flagellin of *B. velezensis* and *B. amyloliquefaciens*. The similarity between BP8-2 and *B. velezensis* was the highest, 85.3383% and 83.8346%, respectively, followed by *B. amyloliquefaciens*, at 83.8346%, 77.6842%, and 73.6842%, respectively, while the similarity with other species of *bacillus* was only 48.4962% and 49.2481%. These results indicated that the antifungal protein BP8-2 identified in the current study was likely to be flagellin.

In order to further verify the ability of flagellin production in Ba168, the hag gene, coding for flagellin was amplified. A fragment of 1002 bp was obtained from Ba168, and it showed 98% similar identity to gene hag (Figure 4B). The amplified gene sequence was translated into amino acid sequence, and the multiple protein sequence alignment analysis was carried out (Figure 5). It was found that the sequences of BP8-2 and the translated flagellin were basically the same, except for the inconsistency of individual amino acid sites. This result indicated that the protein BP8-2 isolated in our study was flagellin. 

## 3. Discussion

In this study, a complete profile of secreted proteins from *B. amyloliquefaciens* Ba168 was constructed by LC-MS/MS. A total of 1155 proteins were identified, and 16 of them were identified as antimicrobial peptides or antimicrobial-activity-related enzymes based on literature reports. One antagonistic protein BP8-2 showed considerable inhibitory activity against apple blue mold and it was identified as flagellin. These results provide a new theoretical basis for the mechanisms underlying the antagonistic activity of biocontrol bacteria *B. amyloliquefaciens* Ba168.

LC-MS/MS combined with the MASCOT database was used to rapidly identify the active proteins in Ba168. The considerable antimicrobial proteins or peptides, with effective biocontrol activities, were secreted from *B. amyloliquefaciens* [29,31,33,34]. However, the conventional antimicrobial peptide research requires several complete separation procedures, including the separation, purification, and identification of isolate substances, following functional research. The mass spectrometry technology offers the possibility of the large-scale rapid identification of proteins without the need for the complete separation of active substances for screening and optimizing the antimicrobial substances production. Caldeira et al. rapidly identified an active lipopeptide in *B. amyloliquefaciens* CCMI 105, by using the LC-ESI-MS methods combined with antifungal tests [35]. Comparing with LC-ESI-MS and other isolation and identification methods, LC-MS/MS can isolate more novel resistant proteins [36]; however, no research has been reported on *B. amyloliquefaciens* by LC-MS/MS. In this study, we adopted proteins that were identified by LC-MS/MS in *B. amyloliquefaciens* Ba168, and new antimicrobial proteins were isolated.

The genome analysis of *B. amyloliquefaciens* FZB42 showed several genes encoding direct synthesis of the polyketides macrolactin, bacillaene, and difficidin [29]. Likewise, our study indicated that the biocontrol effect of Ba168 is the result of a combination of various antimicrobial substances and growth-promoting factors rather than the action of a single substance. In this study, a total of 16 potential antimicrobial-activity-related proteins were identified in Ba168, 10 of which exhibit directed antimicrobial activity and 6 of which are associated with the formation of antimicrobial substances. Among these proteins, surfactin synthase and polyketide synthase are key synthetic enzymes of lipopeptides and polyketides, which are important antimicrobial substances produced by *B. amyloliquefaciens* and exhibit good inhibitory effects against many plant pathogens [37]. Glucanase inhibits the growth of pathogens by hydrolyzing glucan polymers, which are the main component of the cell wall for most pathogenic fungi [38]. Chitin-binding proteins exhibit antifungal activity by binding to monosaccharides and polysaccharides [39]. In addition, the chitin can induce disease resistance in plants and promote plant growth [40]. It is noticed that the synthetic enzymes of fengycin, the important lipopetides, were not identified in Ba168. This result is consistent with previous reports that not all *B. amyloliquefaciens* strains produce all the lipopeptides. For example, *B. amyloliquefaciens* subsp. plantarum B9601-Y2 can produce polyketides and fengycin, but no surfactin has been detected. The underlying reason for this may be that some peptide synthetases involved in the nonribosomal synthesis of surfactin were partially deleted [41]. The findings of these studies indicate that Ba168 can produce a variety of antagonistic proteins for disease prevention and control, which provides a basis for the further application of Ba168.

Flagellin is a structural protein that assembles into the lash-like filament of a bacterial flagellum, which is exclusively found in bacteria [42]. In vertebrates, flagellin is a main component of the flagellar filament. Flagellin is also a specific ligand that stimulates innate immunity through direct interaction with host Toll-like receptor 5 (TLR5). Because flagellin activates the immune response, the development of flagellin as a vaccine adjuvant in subunit vaccines or antigen fusion vaccines has attracted interest [43]. Similarly, flagellin can induce defensive responses in plant cells and has a significant inhibitory effect on a variety of pathogenic fungi [44]. Ren et al. reported that a flagellin-like protein isolated from *Bacillus subtilis* showed antagonistic activity against *Botrytis cinerea* [45]. Fang et al. isolated an antifungal flagellin protein from *B. amyloliquefaciens* using the SDS-PAGE-MS methods [46]. In our work, an antifungal protein named BP8-2 was isolated from *B. amyloliquefaciens* Ba168, and it was identified as a flagellin protein using LC-MS/MS. The gene hag coding for flagellin was amplified from strain Ba168. Protein BP8-2 showed significant inhibitory activity against apple blue mold, inhibited the mycelia growth and spore production in the fungi. Our study provides further evidence that flagellin produced by Ba168 has antimicrobial activity. 

Biocontrol bacteria exhibit their biocontrol capacity predominantly by occupying the infection sites of pathogens on plants, competing with pathogens for water and nutrition, inducing plant systemic resistance, producing metabolites to inhibit the growth of pathogens, as well as competing with pathogens for ecological niches [47]. The BP8-2 protein could lead to abnormal mycelium growth and branching, increased branches, and local swellings. It is assumed that flagellin may have the ability to degrade cell walls. More in-depth mechanisms of the antagonistic activity of flagellin against fungal pathogens need to be studied. More antimicrobial proteins in *B. amyloliquefaciens* Ba168 will be purified in future studies.

## 4. Materials and Methods

### 4.1. Strain and Cultivation Conditions

Strain *Bacillus amyloliquefaciens* Ba168 was submitted to the China General Microbiological Culture Collection Center on 21 August 2012. The preservation number is CGMCC 6462. The strain was stored under freezing conditions at −20 °C and revived 24 h before inoculation in pure inorganic nitrogen medium (20 g/L glucose, 20 g/L (NH)_4_SO_4_, 1 g/L K_2_HPO_4_, 0.5 g/L MgSO_4_, 0.5 g/L NaCl; pH 7.0).

*Penicillium expansum* was isolated from apples and preserved in the Key Laboratory of Molecular Biology for Agriculture, Northwest A&F University. *Penicillium expansum* was cultured on PDA medium at 25 °C for 5~7 days.

### 4.2. Protein Extraction

Ba168 was cultivated at 32 °C and 150 rpm for 72 h, and centrifuged at 12,000× *g* at 4 °C for 5 min. The supernatants were collected, and 313 g ammonium sulfate was added to every 1 L of the supernatants. The mixture was stored at 4 °C for 24 h, and then centrifuged at 12,000× *g*, 4 °C for 10 min to collect proteins. The collected proteins were diluted in 10 mmol Tris-HCl (pH 7.4) buffer, centrifuged at 12,000× *g* for 5 min. The supernatants, which are the protein solutions of Ba168, were transferred to a clean tube and stored at 4 °C for further detection.

### 4.3. Preparation of Proteins for LC-MS/MS

The sample containing 30 μg protein was added into 30 μL SDT buffer (4% SDS, 100 mM DTT, 150 mM Tris-HCl; pH 8.0). Then, the low-molecular-weight components, such as detergent and DTT, were removed by repeated ultrafiltration (Microcon units, 10 kD) using UA buffer (8 M urea, 150 mM Tris-HCl; pH 8.0). It was washed 3 times with 100 μL UA buffer and 2 times with 100 μL 25 mM NH_4_HCO_3_. Then, 100 μL iodoacetamide was added to the filtrate (IAA was 100 mM in UA buffer) and incubated in the dark for 30 min to block the reduction of cysteine residues. Finally, the protein suspension was digested overnight with 4 μg trypsin (Promega, Madison, WI, USA) in 40 μL 25 mM NH_4_HCO_3_ buffer at 37 °C to obtain polypeptides. Sample peptides were desalted on C18 containers (Empore^TM^ SPE Cartridges C18 (standard density), bed I.D. 7 mm, volume 3 mL, Sigma, Saint Louis, MO, USA), centrifuged, and concentrated under vacuum condition, redissolved with 40 μL 0.1% (*v*/*v*) formic acid. The UV spectral density at 280 nm was measured to estimate the peptide content.

### 4.4. LC-MS/MS Analysis

Each protein sample was injected for nano LC-MS/MS analysis. Loading onto the peptide mixture with a reversed-phase trap column (Thermo Scientific Acclaim PepMap100, 100 μm × 2 cm, nanoViperC18, Waltham, MA, USA) connected to a C18 reversed-phase analytical column (Thermo Scientific Easy column, 10 cm length, 75 μm inner diameter, 3 μm resin, Waltham, MA, USA) in buffer A (0.1% formic acid) and separated by buffer B (84% acetonitrile and 0.1% formic acid) in a linear gradient flow rate of 300 nL/min controlled by Intelli Flow technology. Linear gradients of 0–35% buffer B for 50 min and 35–100% buffer B for 5 min were used in 100% buffer B for 5 min.

A Q Exactive mass spectrometer (Thermo Scientific, Waltham, MA, USA) was used to perform LC-MS/MS. This mass spectrometer was coupled to an Easy nLC (Proxeon Biosystems, now Thermo Fisher Scientific, Waltham, MA, USA) for 60 min. The mass spectrometer was operated in positive ion mode. MS data were scanned for the most abundant precursor HCD fragments in a dynamic selection survey (300–1800 *m*/*z*) using a data-dependent top10 method. The automatic gain control (AGC) target was set to 3e6 and the maximum injection time was set to 10 ms. The resolution of the measurement scan was 70,000 at *m*/*z* 200, and the resolution of the HCD spectrum was 17,500 at *m*/*z* 200. The isolation width was 2 *m*/*z*. The normalized collision energy was 30 eV, and the underfill ratio, which specifies the minimum percentage of the target value that could be achieved at maximum fill time, was defined as 0.1%. The instrument was operated in peptide recognition mode.

### 4.5. Data Analysis

Using the MASCOT search engine for searching MS/MS spectra (Matrix Science, London, UK; version 2.2), the search parameters were as follows: database: UniProt; taxonomy: B. amyloliquefaciens (32,598). Antimicrobial proteins were analyzed based on current reports. For protein identification, the following options were used: peptide mass tolerance = 20 ppm, MS/MS tolerance = 0.1 Da, enzyme = trypsin, missed cleavages = 2, fixed modification = carbamidomethyl (C), variable modification = oxidation (M). The mass spectrometry proteomics data have been deposited into the Proteome X change Consortium (http://proteomecentral.proteomexchange.org, accessed on 26 June 2022) via the PRIDE partner repository [48] with the dataset identifier PXD014057.

Multiple sequence alignment analysis of proteins was undertaken in the ClustalW online program (https://www.genome.jp/tools-bin/clustalw, https://www.ebi.ac.uk/Tools/msa/clustalo/, accessed on 26 June 2022).

### 4.6. Purification of the Antifungal Protein

The collected proteins of Ba168 were dissolved in buffer A (10 mmol Tris-HCl) of pH 7.4, then purified using DEAE Bio-sep FF chromatography column using a linear buffer B (0.1–0.7 mol/L NaCl gradient solution). The protein from each peak was collected in a tube. Using *Penicillium expansum* as the test fungus to measure the activity of each peak, it was found that the activity of the 8th peak BP8 was the highest, so the 8th peak was collected and then further purified by gel filtration chromatography using a 2.5 cm × 75 cm Sephadex G-75 column eluted with buffer A. Two peaks were collected and the activity of each peak was measured again. The antifungal activity peak was freeze-dried and suspended in buffer A. 

### 4.7. Inhibitory Effect of the Antifungal Protein BP8-2 on P. expansum In Vitro

The inhibitory effect of protein BP8-2 on *P. expansum* in vitro was determined as Wiggins and Kinkel [49]. Specifically, 10 mL sterile water was added and *P. expansum* was cultured on PDA for 7-days, spores and mycelia were collected by scraping with a sterile loop. Next, 200 μL of the resulting pathogen suspension was sprayed on each PDA plate. Two Oxford cups were then placed symmetrically at the edge of plates, 100–200 μL of BP8-2 protein solution (the protein concentration was 200 µg/mL) was added to each cup. The plates were incubated for 2 days at 25 °C, and the diameter of the fungistatic circle was measured and recorded.

### 4.8. Determination of Minimum Inhibitory Concentration (MIC) of the Antifungal Protein BP8-2 on P. expansum

The MIC for *P. expansum* was tested via the microtiterplate dilution method using 96 well plate method. Specifically, 90 µL *P. expansum* spore suspension was added into 96-well microtitration plate hole for each, then 10 µL of the purified protein BP8-2 with different concentrations in the range of 10–200 µg/mL were added. Sterile water without *P. expansum* was used as a negative control. Sterile water without protein BP8-2 was used as a positive control. After being incubated at 30 °C for 24 h, microbial growth was assessed by measuring the optical density at 600 nm. The MIC was defined as the lowest concentration of flagellin that completely inhibits *P. expansum* growth. 

### 4.9. Control Effect of the Antifungal Protein BP8-2 on P. expansum In Vivo

The control effect of the antifungal protein BP8-2 against *P. expansum* was determined in vivo [50]. After surface disinfection with 0.2% sodium hypochlorite, red Fuji apples were wounded to a depth of 7 mm × 3 mm diameter with a sterile hole punch. 100 μL of protein BP8-2 solution (200 μg/mL) were injected into each hole, 100 μL sterile water injected in each wound was treated as the control. Two hours later, 6 mm disks of *P. expansum* were placed on the wounds, and then sealed with sealing film. All the treated apples were transferred to a biochemical incubator and maintained at a relative humidity of 95% and temperature of 25 °C. Each treatment contained 12 fruits, and the experiment was repeated 3 times. The disease diameter was monitored at 3, 5, 7 and 9 days after inoculation. The prevention and cure effects were calculated as follows: control effect (%) = (1-treatment spot diameter/control spot diameter) × 100.

### 4.10. Identification of the Antifungal Protein BP8-2 and Cloning of Hag GENE in Ba168

The molecular weight of protein BP8-2 was detected by SDS-PAGE, according to the method of Laemmli [51]. Electrophoresis was carried out with 5% stacked gel and 12% running gel. After electrophoresis, the blue band was cutoff and enzymolysized with trypsin. The peptide segment was identified by LC-MS/MS analysis. MS/MS spectra were searched using the MASCOT search engine.

DNA obtained from Ba168 was subjected to PCR for the detection of the hag gene (coding for flagellin). Specific primer pairs (F: 5’-ATGAGAATCAACCACAATATCGC-3’, R: 5’-TTAACCTTTAAGCAATTGAAGAAC-3’) were designed based on the functional gene hag [52]. The amplification system was 20 µL, including 2 µL 10× dream Taq buffer, 0.5 µL dNTPs mixture (10 mM), 1 µL template DNA, 0.2 µL Taq enzyme (0.5 U/mL), 1 µL forward primer (10 µM), 1 µL reverse primer (10 µM) and 14.3 µL ddH2O. Amplification factor: 94 °C, 5 min; (94 °C, 30 s, 55 °C, 30 s, 72 °C, 40 s) for 30 cycle, 72 °C, 7 min, 4 °C termination reaction. PCR products were run according to the method of Kwok et al. [53].

## Figures and Tables

**Table 1:** The 16 potential antimicrobial proteins identified in Ba168.

Project	Protein Name	Protein ID(UniProt)	Gene Name	Molecular Weight (Da)	References
Antimicrobial protein	Oxalate decarboxylase	A0A0G3VD82	*oxdC*	43,574.56	[16]
	Chitin-binding proteins	A0A0D7XZQ5	*gbpA*	22,450.79	[17]
	Flagellin	I2CAR1	*hag*	35,479.84	[18]
	Carboxypeptidase	A0A0K6LSU1	*ykfA*	33,685.03	[19,20]
	Aminopeptidase	I2C7H0	*yqjE*	39,509.56	[21]
	Antimicrobial peptide	I2C153	*lci*	9770.11	[22,23]
	Serine protease	A0A0D7XMJ4	*UZ38_16760*	47,241.97	[24]
	Subtilisin	A0A0K6L9A7	*apr*	39,099.63	[25]
	Leucine dehydrogenase	A0A0G3VBQ2	*ldh*	39,688.26	[26]
	β-glucanase	A0A0D7XPS0	*UZ38_11685*	27,386.34	[27]
Proteins related to the formation of antimicrobial proteins	Surfactin synthase A	I2C187	*srfAA*	402,709.4	[28]
	Polyketide synthase	A0A1J0C866	*BAMY_11440*	573,237.2	[29]
	bacillomycin D synthetase C	H9TE62	*bmyC*	299,764.2	NCBI
	Bacillomycin D synthetase A	H9TE64	*bmyA*	448,739.5	NCBI
	bacilysin biosynthesis protein	A0A142F9I5	*bacA*	27,999.68	[30,31]
	Antilisterial bacteriocin subtilosin biosynthesis protein	A0A0K6M2R3	*albE*	48,507.52	[32]

## Data Availability

Not applicable.

## Sample Availability

Samples of the proteins are available from the authors.
